# Regenerating Family Member 3 Alpha Is Predictive of Mortality Following Emergent Large Vessel Occlusion

**DOI:** 10.3390/ijms25189968

**Published:** 2024-09-16

**Authors:** Madison Sands, Christopher J. McLouth, Jacqueline A. Frank, Benton Maglinger, Nathan Millson, Mais N. Al-Kawaz, Shivani Pahwa, David L. Dornbos, Douglas E. Lukins, Amanda L. Trout, Ann M. Stowe, Justin F. Fraser, Keith R. Pennypacker

**Affiliations:** 1 Department of Neurology, University of Kentucky, Lexington, KY 40506, USA; madison.sands@uky.edu (M.S.); cmclouth@uky.edu (C.J.M.); j.frank@uky.edu (J.A.F.); douglas.lukins@uky.edu (D.E.L.); ann.stowe@uky.edu (A.M.S.); jfr235@uky.edu (J.F.F.); 2 Department of Biostatistics, University of Kentucky, Lexington, KY 40506, USA; 3 Center for Advanced Translational Stroke Science, University of Kentucky, Lexington, KY 40506, USA; david.dornbos@uky.edu (D.L.D.III); a.trout@uky.edu (A.L.T.); 4 BrainCo, Inc., Somerville, MA 02143, USA; bentonmaglinger@gmail.com; 5 Department of Neurosurgery, University of Kentucky, Lexington, KY 40506, USA; nathan.millson@uky.edu (N.M.); mais.alkawaz@uky.edu (M.N.A.-K.); shivani.pahwa@uky.edu (S.P.); 6 Department of Radiology, University of Kentucky, Lexington, KY 40506, USA; 7 Department of Neuroscience, University of Kentucky, Lexington, KY 40506, USA; 8 Department of Otolaryngology, University of Kentucky, Lexington, KY 40506, USA

**Keywords:** neuroinflammation, stroke, statistical modelling

## Abstract

Regenerating Family Member 3 Alpha (REG3A) is an antimicrobial protein secreted by the intestine and pancreas with additional immunomodulatory properties. Previously, we published that REG3A expression in ischemic stroke patient systemic blood, during mechanical thrombectomy (MT), is significantly associated with inflammatory cytokines and patient function on admission. This paper, however, did not investigate post-acute death rates. Therefore, we investigated plasma REG3A protein expression, during MT, in patients (n = 141) that survived or died within the end of the follow-up after MT. Subjects who died had significantly higher systemic plasma REG3A levels at the time of MT compared to survivors (*p* = 0.001). Age, sex, time from last known normal, and admission NIHSS were included as predictors to control for confounding variables and were all examined to determine their association in patient mortality. Logistic regression was used to demonstrate that higher odds of death were associated with increased REG3A levels (*p* = 0.002). REG3A demonstrated acceptable discrimination (AUC (95% CI): 0.669 (0.566–0.772) in predicting mortality. The overall model with age, sex, time from last known normal, and admission NIHSS discriminated well between survivors and those who died (AUC (95% CI): 0.784 (0.703–0.864)). In conclusion, REG3A could be promising as a biomarker to prognosticate stroke outcomes and stratify high-risk groups following acute ischemic stroke.

## 1. Introduction

Stroke is a leading cause of death in the United States, of which 87% are ischemic [1]. Emergent large vessel occlusions (ELVOs) comprise 24–46% of all acute ischemic strokes (AISs) and are a cause of high morbidity and mortality if not urgently treated [2,3]. Mechanical thrombectomy (MT) has advanced the management of ELVO stroke, the most severe type of AIS. ELVO strokes continue to be associated with significant mortality and morbidity [4,5]. Thus, novel prognostic biomarkers and therapeutic targets are much needed to identify and treat such patients.

Regenerating Family Member 3 Alpha (REG3A) is a protein within the REG-family of c-type lectins that are known for their multifunctional roles, such as regulating apoptosis, promoting cellular proliferation and a diversity of antimicrobial functions [6]. REG3A expression has been linked to multiple pathologies, such as cancers, diabetes, pancreatitis and graft-versus-host disease [6,7,8,9,10]. Current evidence supports the utility of increased REG3A as a biomarker of gastrointestinal graft-versus-host disease onset and was predictive of 1-year non-relapse mortality [11]. REG3A levels have also been able to risk-stratify patients based on clinical stage and histological severity during graft-vs-host disease [11]. REG3A was found to attenuate pro-inflammatory cytokine activity and mediate cellular apoptosis by inhibiting pro-apoptotic signalling induced by inflammatory cytokines tumour necrosis factor-alpha (TNFα), IL6, and IL17 [10,11]. These same mediators appeared to reciprocally elevate levels of REG3A, leading to observations of a dose-dependent association between REG3A and the severity of the disease [11].

In the context of ischemic stroke, very little has been published about REG3A. Our previous study demonstrated that systemic arterial blood REG3A levels were associated with increased severity of neurofunctional deficits, via higher NIHSS admission scores [12]. In contrast, REG3A rescued neurons in a rodent model of excitotoxicity demonstrated neuroprotective properties [13]. As far as ELVO stroke subjects, REG3A has yet to be examined in context of mortality, but given its involvement as a biomarker in other disease states, it is a strong candidate for further study as a predictor of stroke outcomes. As such, the aim of this study is to investigate the expression of REG3A in the systemic arterial blood sampled during thrombectomy, assess its relationship to demographic and clinical data, and determine its association with mortality in patients with ELVO strokes. 

## 2. Results

### 2.1. Demographics, Comorbidities, and Stroke Outcomes

One hundred forty-one individuals receiving treatment for ELVO were treated at the University of Kentucky between June 2017 and December 2023 and prospectively enrolled into the study (see Table 1). Individuals who died were older (mean difference = 6.3 years, *p* = 0.015) and were more likely to survive when from non-Appalachian regions (84.8% vs. 67.4%, *p* = 0.027) compared to individuals from Appalachian areas. In an unadjusted analysis of clinical data, individuals who died had higher levels of REG3A, higher infarct and edema volumes, and higher admission and discharge NIHSS. 

### 2.2. REG3A and Mortality

Subjects that died had significantly higher systemic REG3A levels in plasma at the time of MT compared to survivors (*p* = 0.001). Forty-eight patients (34.0%) died during the follow up period after MT. The median time to death was 0.75 months (IQR: 0.20–1.55; Min: 0.03, Max: 49.07). Table 2 presents results in a logistic regression model. REG3A expression was also significantly related to the probability of death. An increase in REG3A of 0.36 on the log_2_ scale was associated with more than a doubling of the odds of death (OR [95% CI]: 2.17 [1.32–3.59], *p* = 0.002). Admission NIHSS score was also associated with the probability of death. A standard deviation increase in NIHSS admission score (SD = 7.0) was associated with an increase in the odds of death (OR [95% CI]: 2.09 [1.31–3.34], *p* = 0.002). No other predictors were significantly related to death. Results did not meaningfully change when time-to-death was limited to within three months. The overall model discriminated well between survivors and those who died (AUC (95% CI): 0.784 (0.703–0.864)). By itself, REG3A demonstrated acceptable discrimination (AUC (95% CI): 0.669 (0.566–0.772)), and the addition of REG3A increased the AUC by 0.062, above and beyond the other covariates in the model.

### 2.3. REG3A and Discharge NIHSS

Table 3 presents results from a linear regression model of REG3A and discharge NIHSS (R^2^ = 0.39). REG3A expression was not significantly related to discharge NIHSS (b (SE) = 0.21, *p* = 0.449). Older individuals and those with longer times from LKN to thrombectomy had significantly higher discharge NIHSS (b (SE) = 0.03 (0.01), *p* = 0.002 and b(SE) = 0.03 (0.01), *p* = 0.046, respectively). Finally, higher admission NIHSS was positively related to discharge NIHSS (b (SE) = 0.10 (0.02), *p* < 0.001). 

## 3. Discussion

Since the advent of MT for ELVO stroke, mortality from AISs has decreased compared to medical management alone [14]. From tissue acquired during MT, groups have been studying the histopathology of the thrombus as well as systemic and intracranial blood to better understand and predict ELVO stroke outcomes [15,16]. Our group has examined samples from patients with ELVO taken at the time of MT showing the proteomic response in systemic and intracranial blood samples [17] and have shown a positive correlation between levels of REG3A and worse NIHSS admission scores [12]. In the current study, we investigated REG3A and its relation to mortality in ELVO subjects treated with MT, and we found patients that died expressed significantly higher levels of REG3A compared to those that survived. While REG3A has been reported to be neuroprotective during brain injury [13], our findings that increased levels of REG3A predict mortality during MT suggest a potentially detrimental role during stroke. This potential paradox could be explained by the elevated levels of REG3A which are being expressed to counter the severe injurious state. This novel finding regarding REG3A indicates a possible biomarker of mortality in ELVO stroke, with an elevation of systemic arterial levels of REG3A in subjects who died versus those who survived. The increased proteomic expression of REG3A was also associated with increased odds of death as an outcome following MT. The examination of receiver operating characteristics demonstrated a significant model with acceptable discriminatory ability [18], with a 66% chance of distinguishing a patient with mortality as an outcome.

For our study, we explored patient demographics and comorbidities that could influence REG3A expression and their effect on mortality. Most collective and individual comorbid states or demographic backgrounds were not different between the two cohorts, and, interestingly, there were no differences between these cohorts regarding the success of intervention measured by CT angiogram scoring and by the perfusion in cerebral infarction (TICI) score. However, the cohort of patients who died and were found to be older had larger infarct volumes, increased severity of neurofunctional deficits, and pre- and post-morbid disability upon hospital admission, discharge, and prior to death. Although not statistically significant, longer infarct times were associated with higher levels of REG3A. As shown previously, higher levels of REG3A were associated with increased severity of neurofunctional deficits on admission [12], NIHSS scores at time of discharge, and increased time between symptom onset and MT. Interestingly, the levels of REG3A were not associated with the extent of vessel perfusion, the reported success of intervention, or the degree of disability at the time of discharge or death. Interestingly, the expression of REG3A was not a significant predictor of patient function after MT as gauged by NIHSS discharge (Table 3). Collectively, REG3A expression was not dependent on co-morbidities or demographics, which supports its use as a biomarker to predict poor outcomes and higher odds of mortality. 

Multiple modalities are used to clinically evaluate stroke severity and clinical outcomes following ELVO. The NIHSS score is widely used upon initial clinical assessment, is associated with larger infarct volumes, and correlates with increased severity of clinical presentation and worse clinical outcomes following ischemic stroke [19]. Several studies that examined the prognostic validity of the NIHSS score demonstrated a discriminative power of 0.72–0.87 compared to the discriminative power of 0.66 for REG3A, as demonstrated in these data [20,21,22]. The mRS score is another tool that can be used to determine intervention efficacy as reflected through the assessment and comparison of an individual’s physical disability [23]. Such predictive biomarkers could improve the identification of high-risk individuals that may benefit from intra-MT therapies, such as intra-arterial therapies (IAT) examined in MR-CLEAN, EXCAP, and SAVER-I studies [24,25]. Thus, the expression of REG3A with these factors could create a predictive model for AIS management. Moreover, REG3A could be a potential therapeutic target for adjuvant treatment with MT.

Limited data have examined REG3A in the context of stroke pathology; however, increased REG3A has previously been identified as a biomarker in inflammation-driven pathologies such as GVHD and gastrointestinal carcinomas [26,27,28]. Likewise, studies have demonstrated a link between different comorbid states and the coordination of immune and inflammatory responses to vessel occlusion [12]. In GVHD pathology, higher levels of REG3A were found to clinically differentiate the severity of rejection disease and predict transplant-related mortality in acute and chronic GVHD [26,27,28]. Moreover, in gastrointestinal carcinomas, elevated levels of REG3A were correlated with increased tumour burden, tumour staging, and lower survival rate in patients with colorectal cancer [29]. In examining inflammatory cytokines and comorbid states, hypertension and obesity in particular were associated with an increase in pro-inflammatory cytokines, such as interleukin 6 (IL6) and interleukin 17 (IL17) [12]. Interestingly, studies have demonstrated a link between REG3A, IL6, and IL17 such that REG3A is directly co-upregulated by these pro-inflammatory cytokines [6,30]. These studies ultimately found that REG3A attenuated pro-inflammatory cytokines at extracellular and intracellular levels [6,30], indicating the possibility that higher levels of REG3A reflect an immuno-inflammatory response following vessel occlusion. Within stroke pathology, inflammatory marker IL6 is associated with a greater incidence of post-stroke cerebral edema and an 18% increased risk of dying during hospitalisation compared to the overall mortality of 3.1% [31,32,33]. Utilising REG3A with additional surrogate outcomes could help to improve our understanding of the pathophysiologic response to AISs and improve the clinical management and identification of high-risk patients.

There are limitations to consider for this study. Here, we measure the levels of REG3A at the time of MT; however, it would be advantageous to know if high levels of REG3A are sustained following MT. If they were sustained following intervention, we could examine post-MT levels as a possible differentiating factor related to mortality between the two cohorts. Another limitation was the sample size of n = 141. Although preliminary, data will continue to be collected and validated as enrolments increase within the BACTRAC study. Lastly, our institution serves primarily eastern Kentucky, which includes Appalachia, well known for health disparities. Approximately 70% of BACTRAC enrollments are reported as residing in Appalachian areas, suggesting that this finding could be unique to our location. Our preliminary findings show a differential inflammatory response between patients from Appalachia and those from non-Appalachian areas (in press, J. Neuroinflammation). With the continued growth of the BACTRAC study, we intend to expand into other regions to diversify our patient population to determine differences in the stroke response dependent on the region.

In conclusion, high levels of REG3A were found to be associated with overall mortality and odds of death occurring in individuals following MT in the setting of ELVO. The premorbid condition and extent of neurofunctional impairment on admission were key factors associated with higher levels of REG3A in the cohort of patients who died. The influence of premorbid and neurofunctional impairment were found to be multifactorial in nature, with a history of prior stroke being associated with more severe premorbid disability and larger infarct volumes being associated with more severe neurofunctional deficits. Interestingly, the success of vessel reperfusion and the initial extent of vessel occlusion were not different between either group and were not associated with the expression levels of REG3A. Thus, we identify REG3A as a novel plasma biomarker for mortality from ELVO after MT, and we additionally suggest its use with other surrogate markers to prognosticate outcomes and mortality in the appropriate clinical context.

## 4. Methods and Materials

The current study received University of Kentucky ethics approval per IRB protocol number 48831 and was not pre-registered, and methods for tissue processing for proteomic analysis have been previously published [15]. This study adopted a prospective study design [34] and was exploratory in nature. No randomisation procedures were applied, and since the study cohort was derived from all enrolled subjects within the University of Kentucky Blood and Clot Thrombectomy Registry and Collaboration (BACTRAC) biobank NCT03153683, no sample size calculation was performed. The image interpreter for measuring infarct and edema volumes was blinded to all patient data except for diagnostic CT-head imaging leading up to mechanical thrombectomy. 

Inclusion criteria were patients undergoing mechanical thrombectomy for large-vessel occlusion stroke. Exclusion criteria included prisoners, pregnant individuals, subjects < 18 years-old, and patients with a past medical history of Moyamoya disease. 

Demographic variables analysed included age and sex, comorbid factors such as hypertension (HTN), hyperlipidemia (HPL), type-2 diabetes (T2DM), and previous stroke. These variables were examined due to their direct relationship to stroke mortality. 

One hundred and forty-one patients were included in this study in which 48 died after the follow-up to MT. Of these, 42 patients (87.5%) died within three months after MT. To better understand other functions related to mortality, we assessed pre- and post-morbid disability, neurocognitive function, procedural success, and other factors reflecting the severity of stroke. NIHSS scores were surveyed upon admission and discharge to reflect the degree of neurofunctional deficit and corresponding severity of stroke. The Thrombolysis in Cerebral Infarction (TICI) scale is commonly used to evaluate the success of recanalisation intervention sequela of AISs [35].

### Statistical Analyses

REG3A levels were analysed as normalised protein expression (NPX), the unit of measurement for protein levels, which is based on a Log2 scale (Olink Proteomics, Boston, MA, USA). Olink has been included in over 1100 publications (https://www.olink.com/ accessed 20 June 2024). The relationship between survival status and demographic and clinical characteristics was assessed using independent sample t-tests and chi-square tests for continuous and categorical variables, respectively. A non-parametric Wilcoxon rank sum test was employed to determine differences in non-normally distributed continuous variables. A multivariable logistic regression model was used to assess the relationship between REG3A expression and death. Age, sex, time from last known normal, and admission NIHSS were included as predictors to control for confounding variables. Regression coefficients from the logistic regression model were exponentiated and presented as odds ratios. Multiple linear regression was used to assess the relationship between REG3A expression and discharge NIHSS. Discharge NIHSS was square root transformed prior to analysis in order to satisfy linear regression assumptions. Data analyses were performed using SAS v 9.4 (SAS Institute Inc., Cary, NC, USA). Missing data were handled using listwise deletion. A *p*-value < 0.05 was used for statistical significance. 

## Figures and Tables

**Table 1:** Demographic, comorbidity, and clinical data for ELVO sample treated by MT.

	Overall Sample	Died	Survived	
	(n = 141)	n = 48	n = 93	*p*-Value
Demographics				
Age in years, mean (SD)	67.1 (14.6)	71.2 (12.7)	64.9 (15.1)	0.015
Sex, n (%) Female	77 (54.6%)	29 (60.4%)	48 (51.6%)	0.320
Race, n (%) White	134 (95.0%)	48 (100%)	86 (92.5%)	0.095
BMI, mean (SD)	28.7 (6.7)	27.8 (6)	29.2 (7)	0.228
Appalachian Residence, n (%)	100 (73.0%)	39 (84.8%)	61 (67.4%)	0.027
Comorbidities				
Hypertension	115 (81.6%)	39 (81.3%)	76 (81.7%)	0.946
Type 2 Diabetes	46 (32.6%)	20 (41.7%)	26 (28.0%)	0.100
Hyperlipidemia	60 (42.6%)	20 (41.7%)	40 (43.0%)	0.878
Previous Stroke	19 (13.5%)	7 (14.6%)	12 (12.9%)	0.782
Clinical Data				
REG3A Expression, Median [IQR]	1.33 [1.17–1.49]	1.41 [1.31–1.65]	1.31 [1.13–1.43]	<0.001
Infarct Volume, median [IQR]	26,940 [9746.5–101,940]	77,720 [27,770–176,600]	14,365 [6595–47,580]	<0.001
Edema Volume, median [IQR]	27,330 [8954.5–107,750]	107,750 [26,270–178,100]	15,065 [5533–48,140]	<0.001
LKN to Thrombectomy in hours, median [IQR]	8.5 [5.2–15.4]	12 [6.1–17]	8.1 [4.7–14.4]	0.063
NIHSS Admission, median [IQR]	17 [11–22]	19 [15–24]	13 [9–20]	<0.001
NIHSS Discharge, median [IQR]	7 [1–15]	17 [12–21]	4 [1–10]	<0.001
TICI Score				0.446
0	4 (2.8%)	2 (4.2%)	2 (2.2%)	
1	3 (2.1%)	0 (0%)	3 (3.2%)	
2A	5 (3.6%)	1 (2.1%)	4 (4.3%)	
2B	44 (31.2%)	17 (35.4%)	27 (29.0%)	
2C	17 (12.1%)	3 (6.3%)	14 (15.1%)	
3	68 (48.2%)	25 (52.1%)	43 (46.2%)	

Note: 1 patient missing BMI, 45 Patients missing edema and infarct volumes, 10 patients missing time from LKN to thrombectomy, 6 missing NIHSS admission, and 19 missing NIHSS discharge.

**Table 2:** Multivariable logistic regression predicting death.

Predictor	Estimate (SE)	Chi-Square	*p*-Value	OR (95% CI)
REG3A	2.14 (0.71)	9.22	0.002	2.17 (1.32–3.59)
AGE	0.03 (0.02)	2.35	0.125	1.29 (0.93–1.77)
Sex ^1^	0.05 (0.23)	0.04	0.833	1.10 (0.45–2.73)
BMI	−0.03 (0.04)	0.79	0.374	0.82 (0.52–1.28)
LKN	0.03 (0.02)	1.83	0.176	1.02 (0.95–1.09)
Admission NIHSS	0.1 (0.03)	9.62	0.002	2.09 (1.31–3.34)

Note. 10 individuals missing LKN, 1 missing BMI, 6 missing Admission NIHSS. 1 Male used as reference. Units for odds ratio interpretation: REG3A = 0.36; Age = 10; BMI = 6.5; LKN = 60 min; Admission NIHSS = 7.0. ^1^ Male used as reference.

**Table 3:** Multiple regression predicting discharge NIHSS.

Predictor	Estimate (SE)	t-Value	*p*-Value
REG3A	0.21 (0.47)	0.46	0.647
AGE	0.03 (0.01)	3.21	0.002
Sex	0.34 (0.26)	1.32	0.189
BMI	−0.03 (0.02)	−1.66	0.101
LKN	0.03 (0.01)	2.02	0.046
Admission NIHSS	0.1 (0.02)	6.1	<0.001

Note. 14 individuals missing discharge NIHSS, 6 missing admission NIHSS, 6 missing LKN, and 5 missing multiple values were removed from analysis. NIHSS discharge scores were square root transformed. Male used as reference.

## Data Availability

The raw data supporting the conclusions of this article will be made available by the authors on request.

## Abbreviations

AIS	acute ischemic stroke
ELVO	Emergent Large Vessel Occlusion
GVHD	graft-versus-host disease
TNFα	tumor necrosis factor alpha
MT	mechanical thrombectomy
BACTRAC	Blood and Clot Thrombectomy Registry and Collaboration
REG3A	regenerating-islet derived protein-3 alpha
PAP1/PAP	Pancreatitis-Associated Protein
TBI	Traumatic Brain Injury
NPX	normalised protein expression
BMI	Body Mass Index
HTN	Hypertension
DM2	Type 2 Diabetes
LKN	last known normal
NIHSS	National Institutes of Health Stroke Score
IL6	Interleukin 6
IL17	Interleukin 17
mRS	Modified Rankin Score
